# Maintaining Family Engagement During Home Visitor Turnover: a Mixed Methods Study of Best Practices

**DOI:** 10.1007/s11121-024-01669-8

**Published:** 2024-04-02

**Authors:** Sarah Kaye, Stephanie Hood, Deborah Cragun, Deborah F. Perry, Paula Cortés Campos, Oluwatosin Ajisope, Annie Davis Schoch

**Affiliations:** 1 Kaye Implementation and Evaluation, Tacoma, USA; 2 University of South Florida College of Public Health, Tampa, USA; 3 Center for Child and Human Development, Georgetown University, 2115 Wisconsin Ave NW, Washington, DC USA

**Keywords:** Evidence-based home visiting, Attrition, Workforce turnover, Configurational comparative methods, Mixed methods

## Abstract

Evidence-based home visiting services (EBHV) are available in states and localities nationwide through the federally-funded Maternal, Infant, and Early Childhood Home Visiting (MIECHV) program. Nevertheless, the anticipated benefits of EBHV, such as improved child developmental outcomes and increased positive parenting practices, may be undermined by the fact that most families withdraw from services earlier than the model developers planned. Prior studies have linked family attrition with staff turnover. The current study used a mixed methods design to investigate the conditions under which families remained active in the home visiting program after their assigned home visitor resigned. Coincidence Analysis revealed that giving families advance notice (at least 1 month) prior to the home visitors’ upcoming resignation or developing a strong positive working alliance with the inheriting home visitor appears to independently make a difference for ongoing family engagement at 3 and 6 months following a staff transition. These findings suggest that emphasizing how staff turnover is managed may mitigate the risk of family withdrawal during these transitions.

**Supplementary Information:**

The online version contains supplementary material available at 10.1007/s11121-024-01669-8.

Evidence-based home visiting (EBHV) programs have been found to achieve positive outcomes for children and families across various domains, including maternal-child health and development and positive parenting (Sama-Miller et al., 2017). In 2010, families’ access to EBHV expanded significantly when Congress authorized a federal home visiting initiative, the Maternal, Infant, and Early Childhood Home Visiting (MIECHV) program, which allocates federal funds for states, territories, and Washington, D.C. to implement one or more EBHV model(s) to serve families in communities with higher risks and obstacles to attaining positive health outcomes (Health Resources and Services Administration HRSA, 2023). While families report high satisfaction levels with EBHV, only half of families remain active after their first year (MIECHV Technical Assistance Coordinating Center [MIECHV TACC], 2015). Across sites and EBHV models, Daro and colleagues (2014) reported that only one-fifth of families received the intended number of visits (as defined by the model developers) in the first 6 months, one-third received 80% of intended visits, and two-thirds received 60% of intended visits.

With such a disconnect between the intended and actual dose of EBHV, research has investigated patterns among families that do and do not remain enrolled. Factors at different ecological levels influence enrollment and retention of families (Cho et al., 2017; McCurdy & Daro, 2004). These include characteristics of the parent and family (client-level), the home visitor (provider-level), the home visiting program (program-level), and neighborhood-level characteristics. Much of the early research focused on identifying client-level factors related to lower rates of engagement and retention in home visiting services, which included maternal age, race/ethnicity, socioeconomic status, parental educational attainment, parental mental health, and linguistic background (Boller et al., 2014; McCurdy & Daro, 2004; MIECHV TACC, 2015). More recently, research has investigated home visitor practices that can predict retention (Fauth et al., 2022). This shift could have significant implications; a study of EBHV retention in Germany reported that 62% of the risk for family attrition could be explained by modifiable factors rather than participant characteristics (Brand & Jungmann, 2014).

In terms of modifiable factors, a growing literature base suggests that the working alliance (i.e., collaborative relationship) between the parent and home visitor is associated with family retention. Building a strong supportive relationship between the home visitor and the family is considered a key mechanism for achieving child and family-level outcomes (Krysik et al., 2008) and has also been linked with greater engagement and participant retention (Beasley et al., 2018; Bower et al., 2020; Kleinman et al., 2023; Torres et al., 2022). Families that withdraw from the program may report a poor “fit” with their home visitor (Holland et al., 2014). In contrast, positive perceptions of the home visitor (using descriptors such as “nice,” “trustworthy,” and “attentive”) were reported by families that were retained (Beasley et al., 2018; Torres et al., 2022). This relationship impacts retention over and above the impact of family satisfaction with services overall (Korfmacher et al., 2007). A recent literature review investigated facilitators and barriers to family engagement in EBHV, where family engagement was broadly conceptualized to incorporate studies of family enrollment, retention, and active participation (Kleinman et al., 2023). In addition to strong relationships, other modifiable facilitators of engagement were flexible scheduling, visit content tailored to families’ goals, and home visitor cultural competence (Kleinman et al., 2023).

Home visitor turnover has been associated with lower family retention (MIECHV TACC, 2015; Rabinovitz et al., 2016). Nationally, over 10% of home visitors resign each year (Lou et al., 2021); local Washington, D.C. data have indicated that this number can be much higher, with up to one-third of home visitors resigning annually (Georgetown University Center for Child and Human Development [GUCCHD], 2016). Holland and colleagues (2014) interviewed families about their reasons for attrition from EBHV, and one reason included being assigned a new home visitor with whom they did not form a strong relationship. A study that reviewed data from over 10,000 families to investigate predictors of attrition described “addressable” attrition as having no visits for over 180 days, having numerous missed appointments, being untraceable, or declining services; the authors reported that parents whose home visitor left the program before the focus child’s first birthday had much higher risk of addressable attrition and ultimately completed fewer home visits with the program (O’Brien et al., 2012). Krysik and colleagues (2008) found that some, but not all, of the families whose home visitors resigned reported difficulty forming a relationship with the new home visitor.

## Research Questions

The evaluation of Washington, D.C.’s MIECHV program has unfolded iteratively and collaboratively in partnership with a single Local Implementing Agency (LIA) and the D.C. Department of Health. The community-engaged evaluation designs have focused on variables of importance to EBHV providers and policymakers, specifically early withdrawal from services and staff turnover. In an earlier qualitative study, families and home visitors were interviewed about how the transitions were handled. Three best practices for transitioning families from a resigning home visitor to an inheriting home visitor were identified during interviews: advance notice of resignation, warm hand-off, and debrief between the resigning and inheriting home visitor about family goals and progress (GUCCHD, 2018). The current study sought to connect those best practices with family decisions regarding whether to stay enrolled after their home visitor resigned. Specifically, the analyses sought to identify minimally necessary and sufficient conditions for families to remain active in voluntary home visiting services when their home visitor leaves. The researchers hypothesized that families that experienced one, all, or any combination of the best transition practices (i.e., advance notice, warm hand-off, and home visitor debrief) might be more likely to develop strong working alliances with the inheriting home visitors and would subsequently remain active in the program at 3- and 6-month post-transition.

## Methods

### Procedures

**Fig. 1:**
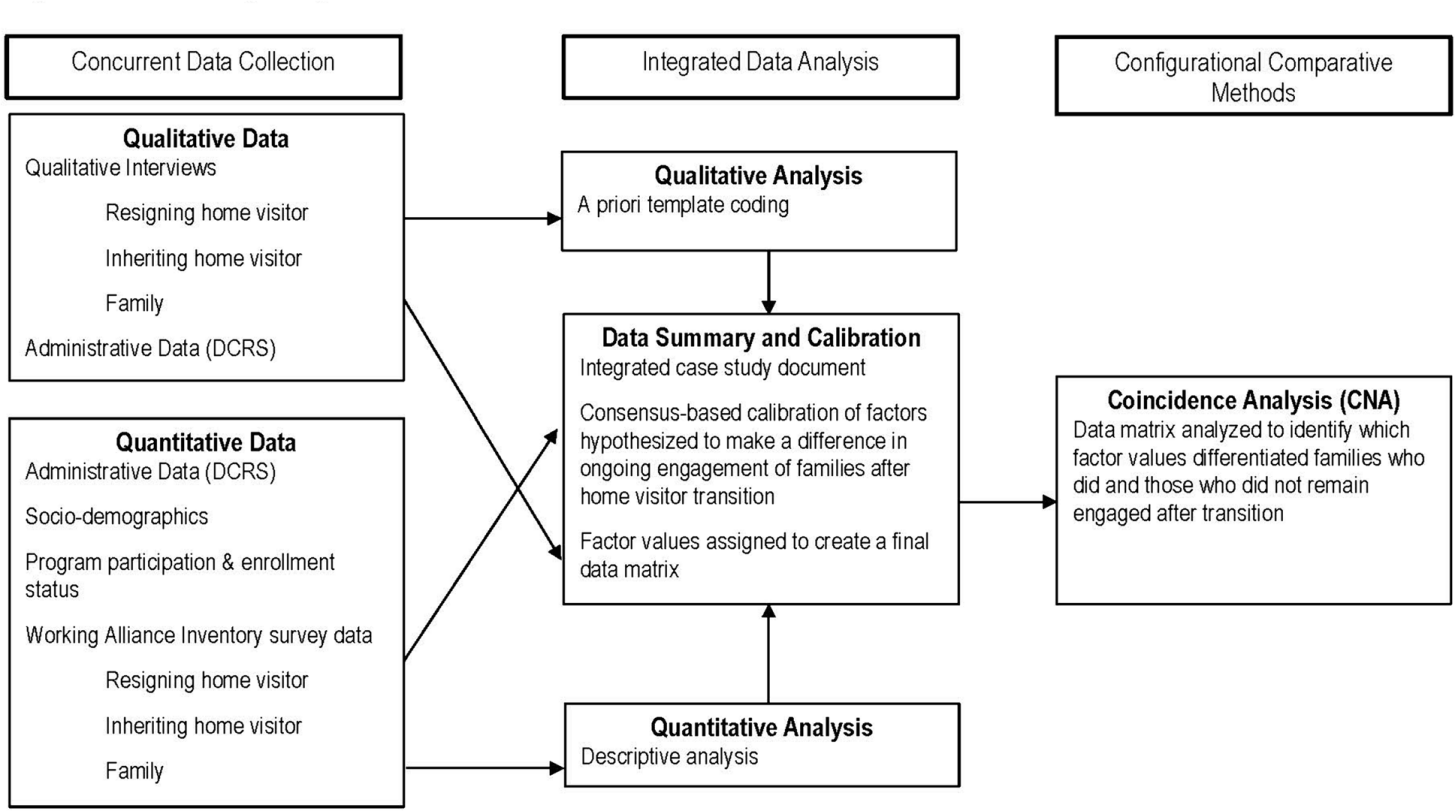
Evaluation study design and method

The current study used a convergent mixed methods evaluation design (Fig. 1).^1^

Briefly, the researchers collected and analyzed quantitative and qualitative data concurrently throughout the 1-year study period to explore how transition practices, working alliances, and other factors influence family retention. The researchers then used Coincidence Analysis (CNA), described in detail below, to determine whether receiving one or more best transition practices consistently made a difference in whether or not families remained active in the home visiting program after experiencing a change in home visitors.

IRB approval for this study was obtained from the D.C. Department of Health and Georgetown University. The evaluation design drew from primary and secondary data sources to integrate and triangulate key perspectives of the resigning home visitors, inheriting home visitors, and families. Primary data collection, including interviews and surveys with home visitors and families, provided significant insight into transition practices and the impact of the transitions. Home visitors signed consent forms to participate in the evaluation at the beginning of the study (or upon employment for those hired during the study period). Working with the LIA, the evaluation team conducted exit interviews and surveys with the resigning home visitors prior to their last day of employment. The evaluation team also invited a sample of families on the resigning home visitors’ caseload to participate in an interview. After receiving approval from the resigning home visitor, the researchers contacted the selected families to request a phone interview. Following informed consent, families that agreed to participate were interviewed at T1 (3 months after their resigning home visitor left). At the time of the interview, families were also asked to complete two working alliance surveys (using the Working Alliance Inventory: Short Form [WAI-SF]: one for their resigning home visitor (retrospective) and one for their inheriting home visitor. Families received $25 for completing the interviews and surveys. Additionally, the inheriting home visitors were contacted at T1 for a brief interview and completed the WAI-SF for the families they inherited.

### Participants

 All home visitors who resigned from their position at the DC-MIECHV-funded Healthy Families America (HFA) program and home visitors who inherited families as part of a colleague’s resignation during 2018–2019 were eligible for the study. Fourteen home visitors participated in the study; they were racially and ethnically diverse college-educated women in their 20 s and 30 s. Descriptive characteristics of home visitors are shown in Table 1. There were two non-mutually exclusive subsamples of home visitors: resigning (*n* = 5) and inheriting (*n* = 12). Three home visitors inherited cases from resigning home visitors before resigning themselves during the study period, so they are included in both the resigning and inheriting home visitor subsamples.

**Table 1:** Characteristics of home visitors

Home visitor characteristics	All home visitors (*n* = 14)	Resigning home visitors^a^ (*n* = 5)	Inheriting home visitors^a^ (*n* = 12)
Spanish-speaking	50%	80%	50%
Length of employment			
< 12 months	50%	60%	50%
12–23 months	21%	40%	17%
24–35 months	14%	0%	17%
> 35 months	14%	0%	17%
Female	100%	100%	100%
Age			
20–29	64%	80%	58%
30–39	29%	20%	33%
40–49	7%	0%	8%
Race/ethnicity^b^			
Black	57%	20%	58%
White	36%	80%	33%
Latinx	14%	20%	17%
Education			
< Bachelor’s degree	7%	0%	8%
Bachelor’s degree	86%	100%	83%
Graduate training or degree	7%	0%	8%
Is a parent	21%	0%	25%

^a^Resigning and inheriting home visitors are not mutually exclusive. Three home visitors who inherited families later resigned during the study period and are also counted as resigning home visitors

^b^Race and ethnicity categories are not mutually exclusive and therefore do not add up to 100%

The researchers were able to conduct interviews with four out of five resigning home visitors and all 12 inheriting home visitors. Of the four resigning home visitors that were interviewed, most were leaving to pursue graduate degrees in nursing or social work, and all notified their supervisors of their intent to leave at least 6 weeks before formally resigning. The fifth home visitor resigned abruptly; thus, the researchers could not interview her. The sample of inheriting home visitors closely resembled the population of home visitors employed by the LIA in age, length of employment, race/ethnicity, and education. The sample of resigning home visitors included a larger proportion of Spanish-speaking home visitors and a smaller proportion of Black home visitors. However, it was similar to the entire population of home visitors in other characteristics (Table 1).

Families enrolled in the HFA program were included in the sampling frame if they received at least one home visit between June 1, 2018, and June 20, 2019, and their home visitor resigned during that time period. Eligibility was determined by examining administrative data from the Data Collection and Reporting System (DCRS), the DC-MIECHV data system. Initially, the researchers proposed to include all of the families on a resigning home visitor’s caseload in the study. However, feedback from a pilot study of these procedures revealed that being asked to report on every family on their caseload during a single exit interview was overly burdensome for the home visitor. Instead, a smaller group of families were purposively selected from resigning home visitors’ caseloads to maximize diversity in: length of enrollment, number of visits received, status in the program, the total number of home visitors that had worked with the family, and service level in the HFA program.^2^ These five sampling criteria were developed based on previous DC-MIECHV evaluation findings and in collaboration with the D.C. Department of Health and the LIA. Using these criteria, the researchers selected eight families for each resigning home visitor, equating to approximately half of each home visitor’s caseload.

At the time of home visitor resignation, the 51 families in the sampling frame had been enrolled for an average of 21 months, with a range from less than 2 months to over 53 months. On average, families had completed just over a quarter of the program. Because this HFA program aims to retain families until the youngest child reaches age five, “completion” was defined as the proportion of the completed program for the youngest child, where the child’s age, in months, at the time of transition was divided by 60 months (i.e., the 5 years of service duration). Of the 32 selected families, about 60% had experienced a transition in home visitor even before the resignation that made them eligible for the study and had, on average, completed 29% of the program. As shown in Table 2, the 32 families selected for the study were similar to the 51 total families that experienced transition on most sociodemographic characteristics, except that families that had a child with a developmental delay were underrepresented in the study sample. Of the 32 families selected for the study, 20 completed an interview in their preferred language of English or Spanish, with response rates higher among English-speaking and unmarried caregivers.

**Table 2:** Characteristics of all families who experienced a transition in home visitor during study period and families in study sample (active and withdrawn)

Family characteristics	All families^a^ (*n* = 51)	Study sample^b^ (*n* = 32)	Retained families (*n* = 25)	Withdrawn families (*n* = 7)
% of caregivers under age 21	6%	3%	4%	0%
Caregiver race/ethnicity				
Black or African American	55%	56%	52%	71%
Latinx	39%	34%	40%	14%
Other	6%	9%	8%	14%
Caregiver primary language				
English	57%	59%	60%	57%
Spanish	35%	31%	36%	14%
Other language	8%	9%	4%	29%
Caregiver working full or part-time	33%	41%	44%	29%
Caregiver married	24%	31%	28%	43%
Caregiver education				
Less than high school	*28%*	*15%*	**16%**	**14%**
High school or GED	*42%*	*46%*	**32%**	**86%**
College or technical training	*30%*	*38%*	**53%**	**0%**
Average number of children	1.2	1.2	1.2	1.1
Child with developmental delay	**10%**	**3%**	4%	0%
Unstable housing	18%	16%	12%	29%
Low-income family	78%	78%	80%	71%

^a^Significant differences between all families and study sample in *italics (p* < *.10)* or bold (*p* < .05)

^b^Significant differences between retained and withdrawn families in bold (*p* < .05)

### Concurrent Data Collection Measures

Semi-structured interview guides included open-ended questions focused on understanding the transition process from the perspectives of families and resigning and inheriting home visitors. Resigning home visitors were asked why they were leaving their jobs, how they prepared families for the next home visitor, and how they prepared the inheriting home visitors to work with the families. Inheriting home visitors were asked whether/how they worked with the resigning home visitor prior to the departure, strategies for building rapport with the new families, and what they perceived the family was gaining from participating in the home visiting program. Families were asked about their relationship with each home visitor, their experiences in the program, their perceived benefits from home visiting services, their transition between home visitors, and how this transition impacted their satisfaction with home visiting services. As appropriate, the researchers who performed interviews would ask additional follow-up questions to probe deeper or to seek clarity about a response. For Spanish-speaking families, the researchers translated the interview protocol into Spanish, making minor adjustments to ensure meaningful and appropriate language use.

All interviews were recorded and transcribed verbatim, redacted of any identifying information, and entered into Atlas ti v7, a qualitative data analysis software. Interviews that were conducted in Spanish were professionally translated and transcribed into English and then reviewed by the bilingual researcher for accuracy.

Working alliance was measured using the Working Alliance Inventory – Short Form (WAI-SF; Santos, 2005), which was adapted for home visiting. The original Working Alliance Inventory (Horvath & Greenberg, 1989) has been well-validated through factor analyses to verify construct validity and subscale structure. Using the WAI-SF, both the resigning and inheriting home visitor were asked to rate their working alliances with the family, and the family was asked to rate their working alliances with resigning and the inheriting home visitor. The WAI-SF includes parallel versions of 12 items that describe ways a home visitor might think or feel about working with the family (e.g., “I am confident in my ability to help the parent,” “The parent and I have built a mutual trust”) and ways a parent might think or feel about working with the home visitor (e.g., “I am confident in my home visitor’s ability to help me,” “My home visitor and I trust one another”). Respondents were asked to rate how frequently statements about working with the home visitor/family were true, on a scale of 1 (*never*) to 7 (*always*). The total WAI-SF score was calculated as an average across all 12 items and ranges from 1 to 7, with higher scores indicating a stronger working alliance.

Administrative data from DCRS provided data to operationalize engagement at T1 and T2 in several ways: the number of visits received, enrollment status (i.e., active or withdrawn), and length of enrollment (i.e., number of months between first and last home visit). Case notes from 6 months before and after the home visitor’s resignation were used to gather dates and summaries of home visitors’ calls and visits with families.

### Integrated Data Summary and Analysis

All interview transcripts, WAI-SF survey results, administrative data including case notes, and case characteristics related to a single family were merged into one integrated document combining qualitative and quantitative data. These integrated case summary documents allowed for a deeper understanding of each family’s transition and were used to summarize and compare responses across the 32 families as part of the subsequent analyses described below.

#### Qualitative Analysis

The researchers took a hybrid approach of thematic analysis (Boyatzis, 1998; Fereday & Muir-Cochrane, 2006) and developed a deductive a priori template of codes, described by Crabtree and Miller (1999), to analyze interviews and case notes. Thematic analysis is not wed to a particular framework or theory but requires deep knowledge of the data to identify patterns arranged into themes (Braun & Clarke, 2006). In this instance, the evaluation questions guided the development of a codebook which was used to analyze the family and home visitor transcripts. The initial code template (informed by a prior qualitative study) included the use of best transition practices, challenges during the transition process, and family impressions of the transition. Themes were then further analyzed to uncover deeper context and meaning of the transition processes as well as the families’ perspectives on home visiting. As is typical with qualitative data, the coding and analysis process was iterative. A single primary coder worked closely with a second researcher to triangulate transition processes described in the interviews with case notes that were entered into DCRS in real time.

#### Quantitative Analysis

Given the small sample size, only descriptive statistical tests were conducted to characterize the sample and to summarize transition practices and working alliances. Several of the inheriting home visitors and families did not feel comfortable completing the WAI-SF, as they had not yet worked with the families long enough to develop a working relationship. During transitions, a supervisor can serve as an interim home visitor while new staff are hired and trained. Inheriting home visitors were encouraged to rate the working alliance based on the limited interaction they had, but the families that opted out of the WAI-SF were assigned a total average score of one (i.e., the lowest possible score) because the qualitative data clearly demonstrated that no working relationship was established.

#### Data Calibration for Use in Coincidence Analysis (CNA)

Working with an expert in CNA, the researchers merged components of the quantitative and qualitative data into a final data set during a process called *calibration* (access the online appendix for details). To guide the process, two of the researchers (SK and SH) developed a calibration rubric by assigning factor values for every case independently, developing consensus, and then obtaining final approval from the study Principal Investig

ator (DFP) and CNA analyst (DC). Factors calibrated for inclusion in CNA were determined with input from program managers at the LIA after reviewing qualitative data summaries (Table 3). Enrollment status was selected as the best measure of engagement for these analyses, and it was dichotomized at T1 and T2. Conditions that were hypothesized by the LIA to make a difference in enrollment status included the receipt of the three best transition practices and the presence of a strong positive working alliance between the inheriting home visitor and the family at T1. Having experienced a second change in home visitor between T1 and T2 was also included due to concerns that this may disrupt ongoing engagement.

**Table 3:** Calibrated variables for Coincidence Analysis of enrollment status

Variable	Original values/sources	Calibrated values		
		0	1	2
Best transition practice				
Warm hand-off	Qualitative interview quotes	Family did not experience it	Family experienced it	NA
Home visitor debrief	Qualitative interview quotes	Family did not experience it	Family experienced it	NA
Advance notice	Qualitative interview quotes	Family did not experience it	Notice from resigning home visitor, less than 1 month prior to resignation	Advance notice from resigning home visitor, at least 1 month prior to resignation
WAI-SF for inheriting home visitor	7-point scale	0 to 4	4 to 6	6 to 7
Family’s enrollment status				
At T1: 3 months post-transition	Administrative data	Family was not active at T1	Family was active at T1	NA
At T2: 6 months post-transition	Administrative data	Family was not active at T2	Family was active at T2	NA
Additional home visitor turnover	Administrative data	Family had home visitor T1 and T2	Family experienced additional home visitor change between T1 and T2	NA

Families that were not active at T1 or T2 could have been withdrawn or lost to follow-up at that timepoint

#### Innovative Analytic Approach: Coincidence Analysis

Unlike traditional regression analyses, Configurational Comparative Methods, like Coincidence Analysis (CNA), use Boolean algebra and set theory to identify necessary and sufficient combinations of conditions that make a difference in the occurrence of an outcome (Whitaker et al., 2020). CNA can be conducted using small to large sample sizes and is not reliant on correlations or statistical power. A key benefit of CNA is its ability to identify if there is more than one “pathway” to achieving an outcome. Additionally, CNA allows for temporal sequencing of factors and outcomes that are measured at different timepoints (details available in the supplemental appendix).

The CNA package in R statistical software was used to conduct multi-value Coincidence Analysis (mv-CNA) using the final data matrix of calibrated factors. Factors were ordered chronologically in groups with the first group including the transition practices (i.e., warm hand-off, home visitor debrief, and advance notice of resignation). The second group of ordered factors was measured at T1, including the working alliance with the inheriting home visitor and enrollment status at 3 months. Lastly, a transition in home visitor occurring between the T1 and T2 enrollment outcomes was ordered before the 6-month endpoint. Both consistency and coverage thresholds were set to 0.90. Briefly, consistency is analogous to positive predictive value and coverage is analogous to sensitivity. Consistency and coverage scores of 1 indicate a perfect fit, but such models may be overfitted. Fit-robustness scores were therefore used to help minimize the risk of overfitting and as an additional model selection tool (Parkkinen & Baumgartner, 2021). Fit robustness was calculated using the frscore package in R with consistency and coverage ranging from 0.75 to 1, going up by increments of 0.05.^3^

## Results

### Enrollment Status at 3 (T1) and 6 Months (T2)

Three months after experiencing a transition in home visitor, 78% of the 32 families (*n* = 25) remained active and continued receiving home visits, while 22% (*n* = 7) withdrew from services. According to administrative data, these families were withdrawn because the program was unable to contact them or to schedule a home visit. Of note, 25% of all the families in the study (*n* = 8) experienced an additional change in home visitor between T1 and T2; three of the eight families that experienced an additional transition are represented within the seven families that withdrew at T1.

Six months after experiencing the original transition in home visitor, 81% (*n* = 26) were actively enrolled. According to guidelines for HFA, families who miss a number of scheduled home visits and/or become hard to reach may stay enrolled but are placed on “creative outreach” for a period of time. Notably, 23 of the 25 families (92%) who were active at T1 remained active at T2. Administrative data for the two families that withdrew between T1 and T2 indicated that one family “aged out” and the other was unable to be contacted by the program. Neither of these families was among the eight who experienced an additional transition in home visitor between T1 and T2. Of note, all eight of the families that experienced an additional transition between T1 and T2 remained active at T2. Five of these families remained active at both T1 and T2, while three families that withdrew at T1 later re-engaged by T2. As a result, the final number of families that were active in the program was slightly higher at T2 than at T1 (81% versus 78%).

Families received best transition practices at different rates. Specifically, 41% of families (*n* = 13) received advance notice from their home visitor at least 1 month prior to the resignation, while 31% (*n* = 10) were notified of the resignation but were told less than 1 month before; 28% (*n* = 9) were not informed of the resignation until their home visitor’s last day. While only 25% (*n* = 8) of the families had a warm hand-off (which included a joint visit with the resigning and inheriting home visitor together with the family), the resigning and inheriting home visitors were able to debrief about the family’s progress and goals in 53% of the cases. A total of 19% of families (*n* = 6) had all three best transition practices, 9% of families (*n* = 3) had two of the best practices, 31% (*n* = 10) of families had one of the best practices, and the remaining 34% of families (*n* = 11) had none. Most of the families who received no best practices had the same home visitor who resigned abruptly without notice.

### CNA Results and Model Selection

**Fig. 2:**
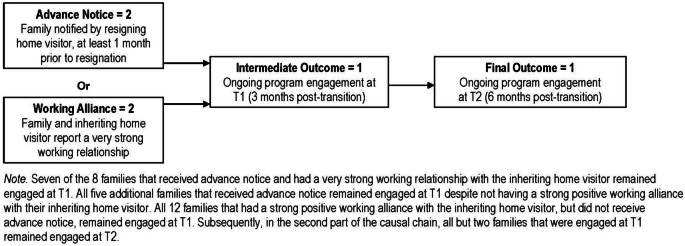
Most robust model from Coincidence Analysis showing conditions that make a difference for ongoing engagement post-transition at 3 (T1) and 6 (T2) months

Results of the initial CNA analysis identified 15 complex models that met or exceeded the consistency and coverage threshold of 0.9. One model “stood out” as having the highest overall consistency (0.93) and coverage (0.96). However, subsequent robustness analyses (which varied consistency and coverage thresholds) revealed that the initial “stand out” model was only the second most robust model (relative fit robustness of 0.67). On the other hand, the most robust model (relative fit robustness of 1.0) had lower consistency (0.92) and coverage (0.88). The most robust model (illustrated in Fig. 2) and the “stand out” model consist of all the same components, but the initial “stand out” model is slightly more complex. For the sake of parsimony, the most robust model will be described below.

The robust model revealed two independent pathways that are consistent with ongoing engagement at T1: (1) having received advance notice of the upcoming home visitor resignation (at least 1 month prior to the transition), or (2) having a strong positive working alliance with the inheriting home visitor at T1. Although the presence of only one of these two variables was minimally necessary for ongoing engagement, eight families had both at least 1-month notice from the resigning home visitor as well as a strong positive working alliance with the inheriting home visitor. Seven of these eight families remained active at T1, and one was inconsistent with the model. Five additional families that received advance notice remained active at T1 despite not having a strong positive working alliance with their inheriting home visitor. Additionally, all 12 families that had a strong positive working alliance with the inheriting home visitor, but did not receive advance notice, remained active at T1. Only one family remained active even though they did not experience advance notice and did not have a strong positive working alliance. This family’s outcome could not be explained by this model (i.e., it is not covered).

The model also identified what is referred to as a *causal chain* whereby the presence of either one of two difference-making conditions described above resulted in active enrollment at T1, which subsequently resulted in continued enrollment at T2. All but two families that were active at T1 remained active at T2. The three families that were not active at T1, but then re-engaged between T1 and T2, remain unexplained in the most robust model.

Interestingly, the initial “stand out” model (shown in the supplemental appendix) contains an additional path whereby a change in home visitor leads to re-engagement at 6 months. This additional path covered the three additional cases and resulted in higher coverage and slightly higher consistency as compared to the most robust model. However, the additional path to the 6-month outcome ran contrary to what was originally anticipated, none of the available qualitative data supported the idea that a second change in home visitor led to re-engagement, and there was concern that the “stand out” model could have been overfitted.

## Discussion

To the researchers’ knowledge, this is the first study to use an integrated mixed methods approach with Coincidence Analysis (CNA) to identify factors that make a difference for ongoing family engagement in home visiting programs following a transition between home visitors. While many studies have documented the high rates of family attrition from EBHV and the increased risk for attrition when there is home visitor turnover (MIECHV TACC, 2015), few have unpacked what actions home visitors and programs can take to reduce attrition. Since research supports the importance of the home visitor-family relationship as well as program flexibility and tailoring in retention and outcomes (Bower et al., 2020; Kleinman et al., 2023), the current study sought to understand how to retain families after home visitor turnover by analyzing the role of program practices and relationships.

Deeply understanding transitions from multiple perspectives can inform which practices seem to support families through a change in home visitor so they remain engaged in the home visiting program. Contrary to expectations, most families whose home visitor resigned remained active at T1 and T2. Higher than expected levels of ongoing engagement in this study may be a function of work the DC-MIECHV LIA had done to ensure best transition practices were implemented during transitions. Underscoring this, when these data were shared with the LIA, program leaders indicated that upon enrollment, they emphasize that families are joining a home visiting program, rather than being assigned to an individual home visitor.

According to these results, one condition that appeared to lead to ongoing engagement was having advance notice of the change in staff. This finding suggests that home visitor transitions, if managed well by the program, may not be as large a factor in predicting family attrition as initially thought. In their integrative review, Bower et al. (2020) found only two studies that focused on the relationship between home visitor turnover and parent involvement in MIECHV programs; while both studies suggested that turnover was a barrier to retention, the current study suggests that transition practices (specifically advance notice) may help mitigate this barrier.

Interestingly, 12 families remained active even in the absence of advance notice. These families all had a strong working alliance with the inheriting home visitor, which seemed to act as a second independent condition to mitigate any negative impact of home visitor turnover. Torres et al. (2022) also examined the role that a strong therapeutic alliance played in retention of families in an infant mental health home visiting model in Michigan. Overall, a higher score on the Scale to Assess Therapeutic Relationships (STAR; McGuire-Sneickus et al., 2007) predicted longer duration in services; families with lower scores were more likely to drop out 3 months after enrollment. This same research team used survival analysis to examine the independent and cumulative effects of a range of socio-demographic and mental health factors on retention (Jester et al., 2023). Their findings mirror others who report shorter retention predicted by a composite variable reflecting socio-demographic factors, but longer engagement for families with higher mental health burdens. They hypothesize that this finding might be driven in part by the fact that their home visitors were licensed mental health professionals, in contrast with many other EBHV programs. Their work underscores the need to jointly consider characteristics of the enrolled clients, the home visitors, and relational variables such as the therapeutic alliance.

### Limitations and Strengths

As with many studies in this field, much of the data relied on accurate recall and reporting of information, which inherently contains error. In this case, home visitors reported the length of advance notice they provided to LIA leadership (not externally validated) and families reported retrospectively on their relationships with the home visitors who resigned 3 months prior. The researchers originally proposed to have two interviews with families: one when their home visitor resigned and a second one 3 months later, to parallel the procedure followed for home visitors. However, during the pilot, the LIA team expressed concern about interviewing families during the transition. While recall lapses or social desirability may have affected responses, the triangulated approach to data collection may have mitigated the impact of these biases on outcomes. Importantly, in a community-engaged project, data collection decisions were made in collaboration with LIA staff to minimize burden and distress to participants (both home visitors and families).

In respecting the LIA’s request not to overburden resigning staff with having to complete the Working Alliance Inventory with all families on their respective caseload, potential bias may have been introduced into the study sample. Families were purposively sampled to increase the diversity on the variables listed earlier in the Methods section; but that may have led to differences in other characteristics, such as levels of education, percentage of married participants, those working full-time, and percentage of families that have a child with a developmental delay. In addition, the administrative database does not track how many changes in home visitors a family has experienced since enrollment, so it was not possible to purposively sample for that experience, which might have been a “difference-maker.” Those families who had already successfully navigated a prior transition might be more resilient in the face of a subsequent transition.

The research team selected comparative configurational methods, and specifically CNA, because the DC-MIECHV program has only one LIA, and therefore sample sizes are always underpowered for traditional statistical methods. Comparative configurational methods can be successful with sample sizes as small as 10–15 cases, so this study’s sample of 25 families was appropriate. This research also relied on a mixed methods design, which created deep knowledge of the cases and is a requirement for CCMs. While there was only one coder for the qualitative analyses—which would be a limitation in a purely qualitative study—these data were combined with other quantitative data in the calibration process of CNA. And finally, these methods allow researchers to identify patterns in the data that are referred to as causal chains; however, these are not the same type of phenomenon as causal assertions made from a randomized controlled trial.

## Conclusion

The current study used a small urban sample and an innovative approach to data analysis to understand the conditions under which staff turnover does and does not result in families exiting home visiting services. The study drew from primary and secondary sources to integrate and triangulate key perspectives of the transition process when home visitors resign. This approach resulted in a rich description of transition practices, facilitating an in-depth understanding of the complex process of transitioning families from one home visitor to another. Since the study relied on data from a single program at a single agency, additional research is needed in other settings to explore the relevance of these practices for other models and populations.

### Supplementary Information

Below is the link to the electronic supplementary material.

Supplementary file1 (DOCX 856 KB)

## Footnotes

1 Access the supplemental methods and results appendix available online for details.

2 Each family is assigned to a service level in HFA, which prescribes the recommended number of home visits per week based on the family’s level of risk.

3 Additional information about the processes involved in the Coincidence Analysis is included in the online appendix.
