# Supplementary material for: Maintaining Family Engagement During Home Visitor Turnover: a Mixed Methods Study of Best Practices
Source: Prev Sci. 2024 Apr 2;25(3):470–80. doi: 10.1007/s11121-024-01669-8 (PMC11093809; doi:10.1007/s11121-024-01669-8)
Supplement: Supplementary file 1 — Supplementary file1 (DOCX 856 KB) [file 11121_2024_1669_MOESM1_ESM.docx]

**Appendix: Supplemental Methods and Results Details**

***Measures***

Semi-structured interview guides included open-ended questions focused on understanding the transition process from varying perspectives. Exit interviews explored reasons why home visitors resigned or were reassigned, how home visitors prepared families for the next home visitor, and how they prepared other home visitors to take on specific families. Questions asked of the inheriting home visitors focused on how they worked with the resigning home visitor prior to their departure, strategies for building rapport with the new families, and what they perceived the family was getting out of participation in the home visiting program. Families were asked about the relationship they had with each home visitor, and how/if this transition impacted their experience with home visiting services. As appropriate, interviewers from the evaluation team would ask additional follow-up questions to probe deeper into a response or to seek clarity about a response.

For families where English was not the primary language in the home, the evaluation team translated the interview protocol into Spanish, making minor adjustments to ensure meaningful and appropriate language was used. Interviews were conducted in Spanish, recorded and professionally translated and transcribed in English, then reviewed by the evaluation team for accuracy.

In order to understand working alliance as it relates directly to home visiting programs the Working Alliance Inventory – Short Form (WAI-SF) was used. It includes 12 items rated on a 7-point scale, where 1=Never and 7=Always. The total WAI-SF score is calculated as an average across all 12 items and ranges from 1 to 7.

*Data analysis*

This study used a convergent mixed methods evaluation design that involved concurrent data collection, merged integration, and embedded data analysis (Figure 1). Specifically, the evaluation team collected and analyzed quantitative and qualitative data concurrently, throughout the one-year study period to explore how transition practices, working alliances, and other factors influence family retention. The evaluation design drew from primary and secondary data sources to integrate and triangulate key perspectives of the resigning home visitors, inheriting home visitors, and families. The selected methods provided a rich description of transition practices and facilitated an in-depth understanding of the complex process where families transition from one home visitor to another.

**Figure 1.** Evaluation study and design methods


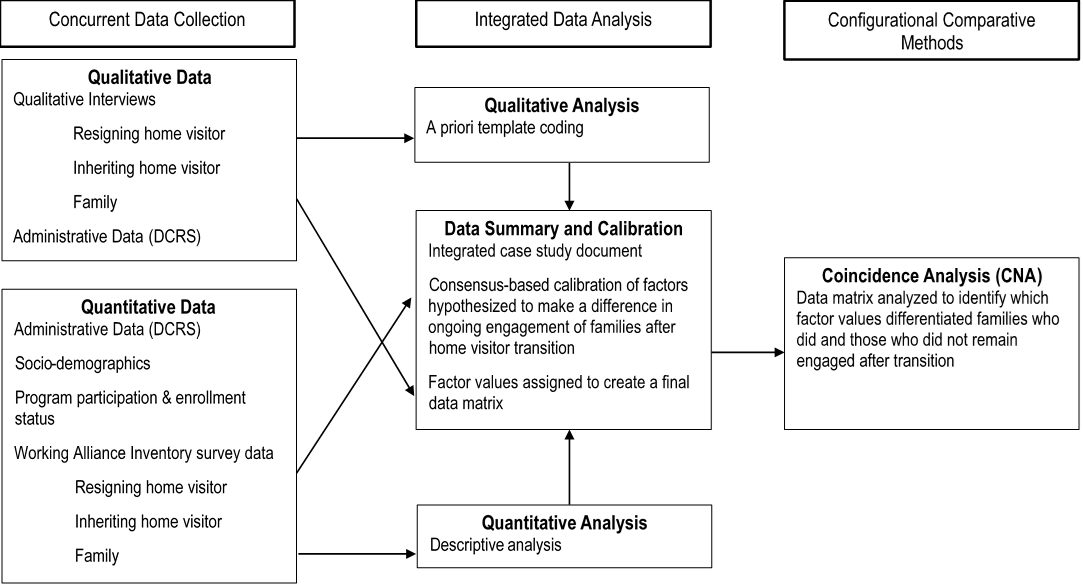


All interviews were recorded and transcribed verbatim, identifiers were redacted, and transcripts entered into qualitative software program Atlas ti v7. The evaluation team took a hybrid approach of thematic analysis and a deductive a priori template of codes described by Crabtree and Miller. In this instance, the evaluation questions guided the development of a code template. The initial code template included use of best transition practices, challenges within the transition process, and family impressions of transition. Themes were then further analyzed, to infer deeper context and meaning of the transition processes and families’ perspectives on home visiting. As is typical with qualitative data, the process was iterative in nature. The primary coder worked in close communication with another member of the research team to triangulate processes described in the interviews with data entered into an established administrative data system. Data were intentionally collected so there are three perspectives of the same event. All transcripts related to one family were merged into one integrated document to identify inconsistencies and alignment between interviews. This allowed for ease of triangulation and readability when coding.

***Data integration, calibration, and factor selection***

Evaluators developed integrated case study documents that merged quantitative and qualitative data from DCRS, case notes for the three months preceding and three months following resignation, visit and home visitor history, and interview transcripts into a single file for each case. To guide the process, two researchers developed a calibration rubric by assigning factor values for every case independently, developing consensus, and then attaining final approval by the study Principal Investigator and the researcher who conducted CNA. The process of calibration was necessary to prepare data for use in coincidence analysis (CNA) which is a type of configurational comparative method (CCM) described more in the section below.

Factors that were ultimately included in CNA were selected by the evaluation team with input from Program Managers at the LIA who reviewed qualitative data summaries. During this process, “transition summary” was excluded as a best practice in the analysis because none of the inheriting home visitors mentioned this as being helpful during their interviews and it is uncertain whether inheriting home visitors always review the transition summary document. To calibrate select factors, each of the other four transition best practices were assigned a score of 1 if the family experienced that transition practice and 0 if they did not. The researcher conducting CNA noted that families given advanced notice were a subset of those who were notified by the resigning home visitor. Therefore, these two transition practices were combined into a single multi-value factor as follows: families given advance notification by the resigning home visitor were assigned a value of 2, those notified by the resigning home visitor but not in advance were assigned a 1, and those families who were not notified by the resigning home visitor were assigned a value of 0.

Inheriting home visitor scores on the WAI-SF were used because this measure had fewer missing values and they were generally concordant with WAI-SF scores completed by the families. To calibrate the working alliance, the team assigned a value of 2 to those with a WAI-SF score above 6 on the 7-point scale to indicate a strong positive working alliance, a WAI-SF between 4-6 was assigned a value of 1 to indicate a weakly positive working alliance and any score of 4 and lower was assigned a 0 value to indicate the absence of a positive working alliance.

Ongoing engagement in the program at 3-months and 6-months after the home visitor resigned were each calibrated as 1 if the family had ongoing engagement at the respective time and 0 for families that had dropped out of the home visiting program at that time. A few families who were no longer engaged at the 3-month follow-up became engaged again and these families were assigned a value of 0 for 3-month engagement and a value of 1 for 6-month engagement. Finally, having an additional home visitor change was calibrated as 1 if the family experienced another change in home visitor between the 3 and 6-month follow-ups and 0 if there was no additional change in home visitors.

**Table 1.** Calibrated factor values used in CNA

| Factors | Calibrated Factor Values |
| --- | --- |
| ^a^Warm handoff (WH) | Families experienced WH=1; did not experience WH=0 |
| ^a^Debrief (D) | Home visitors completed debrief = 1; No debrief completed =0 |
| ^b^Notification (N) | Families given advance notification by resigning home visitor=2; notified by the resigning home visitor but not in advance= 1; not notified in advance by the resigning home visitor = 0 |
| Working alliance (WA) between family and inheriting home visitors | Strong positive WA = 2 (score above 6 on 7 point scale); weakly positive working alliance = 1 (score between 4-6); absence of a positive working alliance= 0 (score of 4 or below) |
| On-going engagement at 3 months | Active or on creative outreach (3 months after resigning home visitor) = 1; left program before 3 months = 0 |
| Another change in home visitor | Family experienced another change in home visitor between the 3 and 6-month follow-ups = 1; no additional change in home visitor during that time = 0 |
| On-going engagement at 6 months | Active or on creative outreach 6 months after resigning home visitor) = 1; left program between 3-6 months = 0 |

^a^Two of the transition best practices were assigned a score of 1 if the family experienced that transition practice and 0 if they did not.

^b^All families given advanced notice were notified by the resigning home visitor. Due to this subset relationship, these two transition practices were combined into a single multi-value factor.

***Coincidence analysis (CNA)***

CNA is a unique type of analysis that can identify patterns of one or more conditions that make a difference for one or more outcomes of interest. CNA is useful for evaluating complexities inherent in real world settings. For example, CNA can identify if there is more than one “pathway” to achieving an outcome. CNA can also find two or more conditions that together may lead to an outcome even though each condition alone is insufficient for the outcome. Finally, CNA can uncover “causal chains” where a condition leads to an intermediate outcome, which then leads to a distal outcome.

CNA is advantageous over path analysis for two main reasons. Path analysis requires larger sample sizes than are often available when studying family or organizational units and it also requires the researcher to specify the structure of the model. In contrast, CNA can be conducted with small to large sample sizes; and the CNA algorithm uses Boolean algebra (rather than correlations) to build models from the ground up that fit the underlying data at specified consistency and coverage thresholds. Although researchers do not pre-specify the model structure, they can constrain certain models by specifying an outcome or an ordering in CNA.

We used the CNA package in R statistical software and raw data matrix to conduct multi-value coincidence analysis (mv-CNA). Unlike the raw data matrix which consists of 32 rows of factor and outcome values (one for each family), the configurational table combines families that share the same configuration of conditions and outcome values into a single row to reveal a simplified visual (see Table 2 below).

**Table 2.** Table of Case Configurations


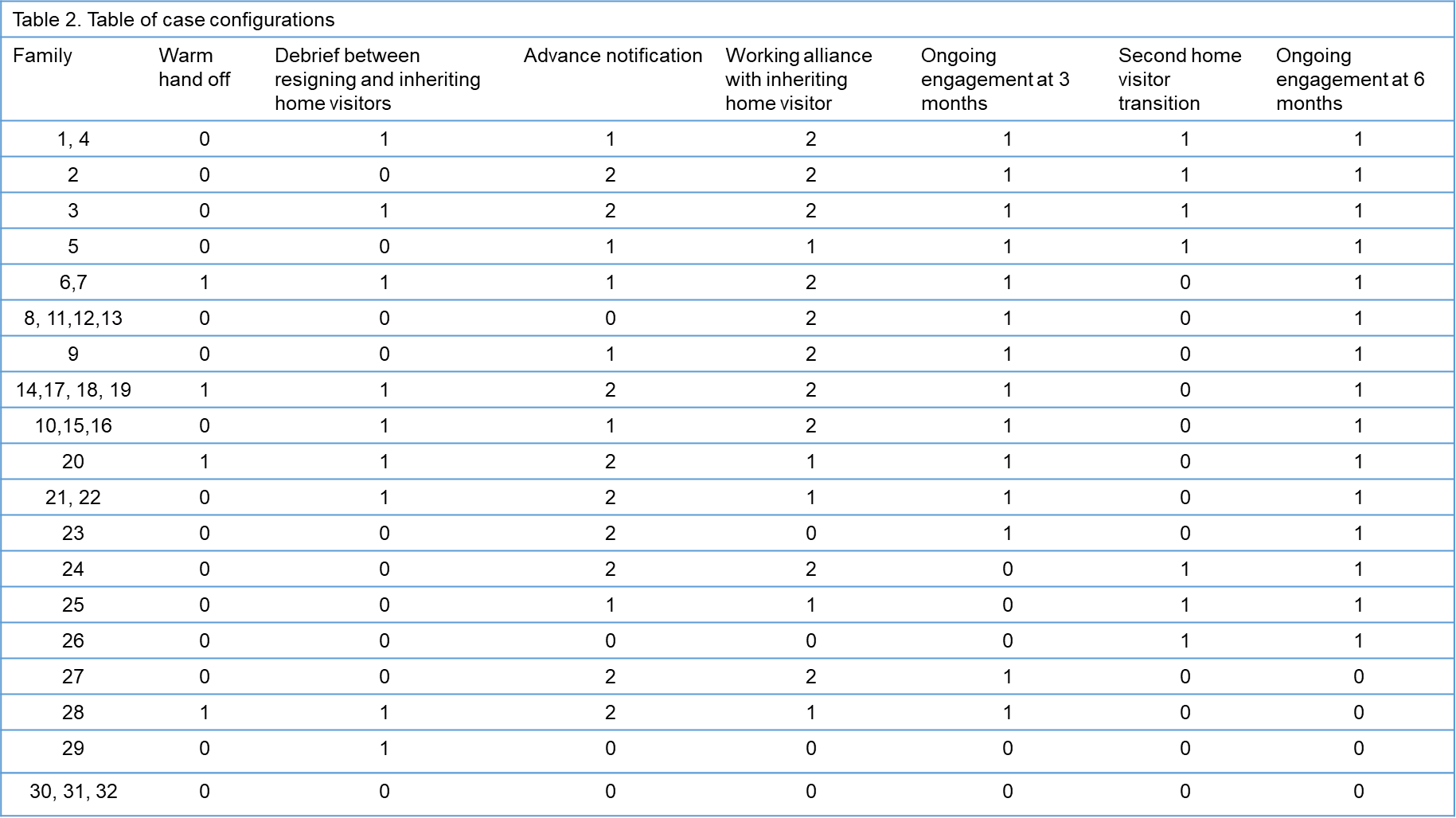


The CNA algorithm compares these configurations of factor values (i.e., conditions) to determine which conditions are minimally sufficient and necessary for the outcome. When conducting CNA, we ordered factors chronologically in groups so that the algorithm could identify “causal chains” if substantiated by the data (see Figure 2 below). Causal chains occur when one or more factor values are Boolean difference makers for another factor value (i.e., an intermediate outcome value) which, in turn, makes a difference for the final outcome value. The first group of factors included: 1) warm handoff; 2) home visitor debrief; and 3) notification by the resigning home visitor. The second group of ordered factors were measured at 3 months after the transition and included the working alliance with the inheriting home visitor and ongoing engagement at 3 months. Lastly, a change in home visitor between the 3-month and 6-month follow-ups was ordered before the final outcome.

**Figure 2.** Ordering of the calibrated data used in CNA.


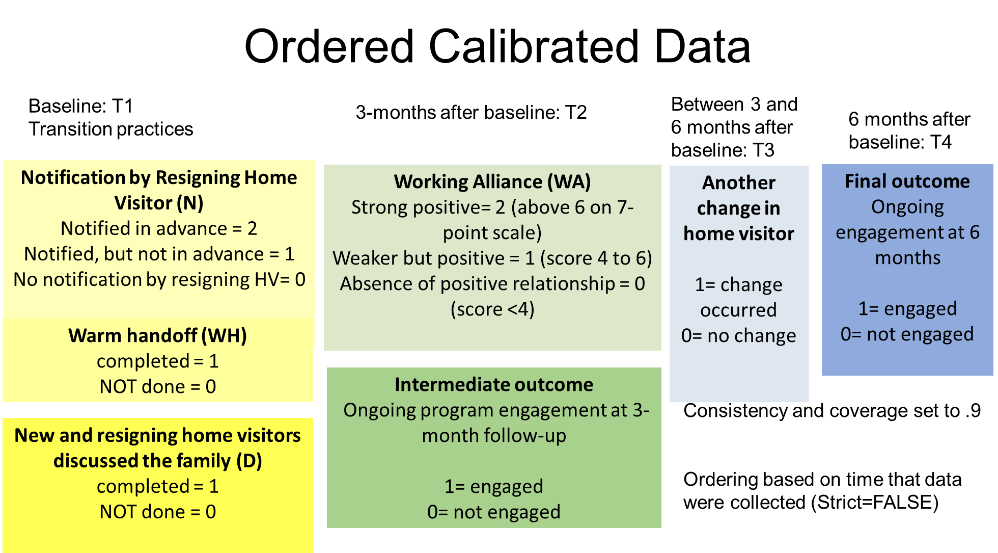


As part of the ordering argument, ‘strict’ was set to FALSE so that factors within each group could impact each other or they could impact any of the downstream factors/outcomes (but downstream factors could not impact any of the upstream factors that were measured at an earlier time). When initially running CNA, the consistency and coverage thresholds were set to .90. Consistency and coverage are used by the CNA computer algorithm to build redundancy free models from the bottom up that fit with the underlying data structure. Consistency and coverage also serve as measures of model fit and range from 0 (lowest possible value) to 1 (highest possible value). Consistency is analogous to positive predictive value. In this study, the numerator used for calculating consistency is the number of families that have both the outcome value (i.e., ongoing engagement) and have a configuration of conditions that is consistent with the model solution (i.e., have a configuration of condition(s) in at least one of the “causal paths” to the outcome value). This numerator is then divided by the total number of cases that are consistent with the solution. Coverage is analogous to sensitivity and is calculated by taking the same numerator as before but dividing it by the number of families that have the outcome value (i.e., ongoing engagement). The mv-CNA software calculates consistency and coverage separately for each path leading to the intermediate and final outcome value, after which the lowest of these scores is used to represent the overall consistency and coverage of the complex model (i.e., model containing all paths to all outcome values).

There are often several distinct models that explain the outcome(s) and that fit quite well with the underlying data structure (i.e., multiple models can have relatively high consistency and coverage). It is important to note that different models are not the same as different pathways within the same model. Different models may share overlapping conditions and pathways but will have some differences. Models that do not contradict (but have either extra or missing paths/conditions demonstrate supermodel or submodel relationships and these can be distinguished from models that have contradictory paths or conditions.

When there are multiple models we cannot be sure which one is “best”. Historically, the model with the highest coverage and consistency was often selected and presented. However, this is not recommended as best practice now due to concerns about overfitting (Parkkinen & Baumgartner, 2020). An overfit model means the model may contain one or more conditions in the solution that are not actually making a difference for the outcome (i.e., analogous to false positive findings).

Since multiple models are often output by CNA, we used fit robustness scores and discussion with community advisors to help select among the models (Parkkinen & Baumgartner, 2020). Relative fit robustness scores were calculated using the first version of the frscore package in R with consistency and coverage ranging from .75 to 1, going up by increments of .05. The parameters were set as follows: ‘scoretype’ = ‘full’, ‘score normalization’ = truemax, and ‘maxsols’ = 300. Essentially the frscore package uses the CNA algorithm to build models that meet varying thresholds of consistency and coverage and then compares these models. A model with more submodel and supermodel relationships will be scored higher on fit robustness as such a model should be less likely to be either overfit or underfit (i.e., missing conditions or paths).

***Results and model selection***

Results of the initial multi-value CNA identified fifteen models that met the consistency and coverage threshold of .9. The model with the highest consistency and coverage was originally preferred as a “stand out” model and is shown below in Figure 3.


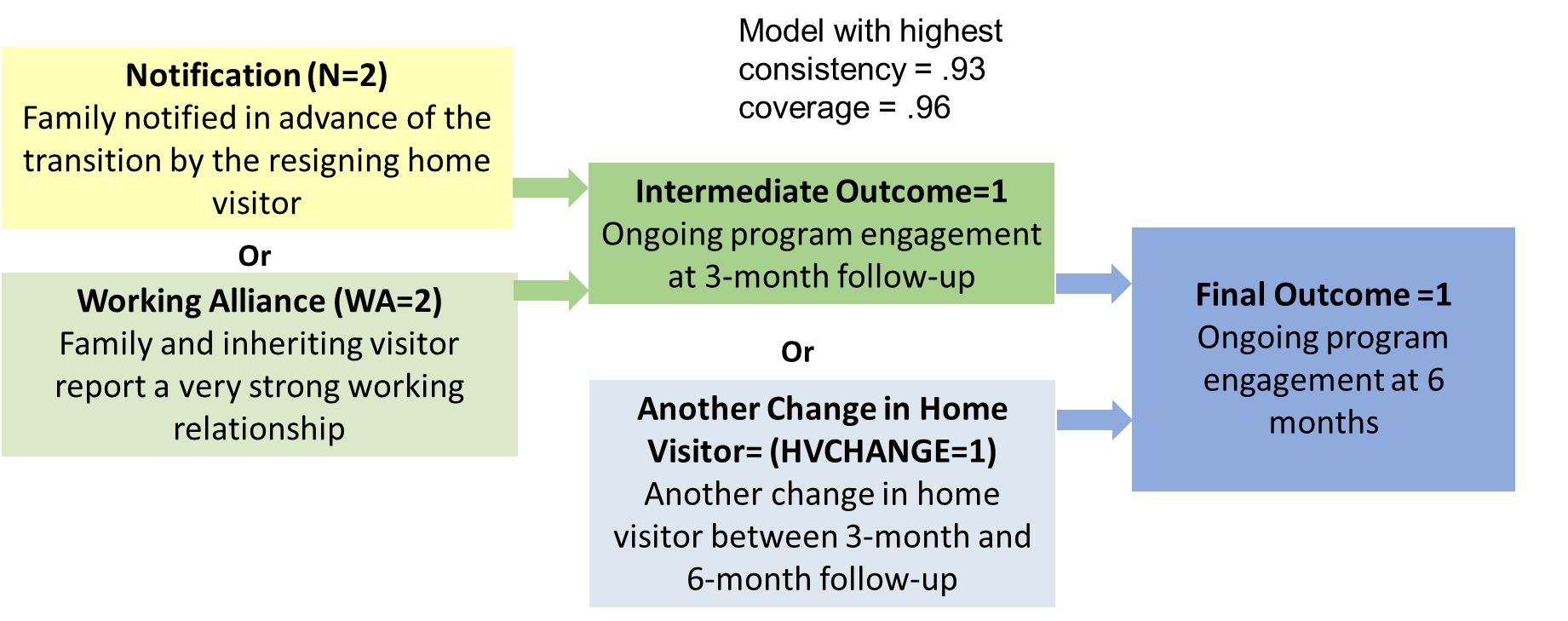
**Figure 3.** Initial “stand out” model among those with consistency and coverage of .9 and above.

After running CNA using frscore, the single most robust model (normative fit robustness of 1.0) was selected as the focus for the paper even though it had lower coverage of .88 (see Figure 4 below).

**Figure 4.** “Most robust” model that was selected because it was less likely to be overfit than the “stand out” model


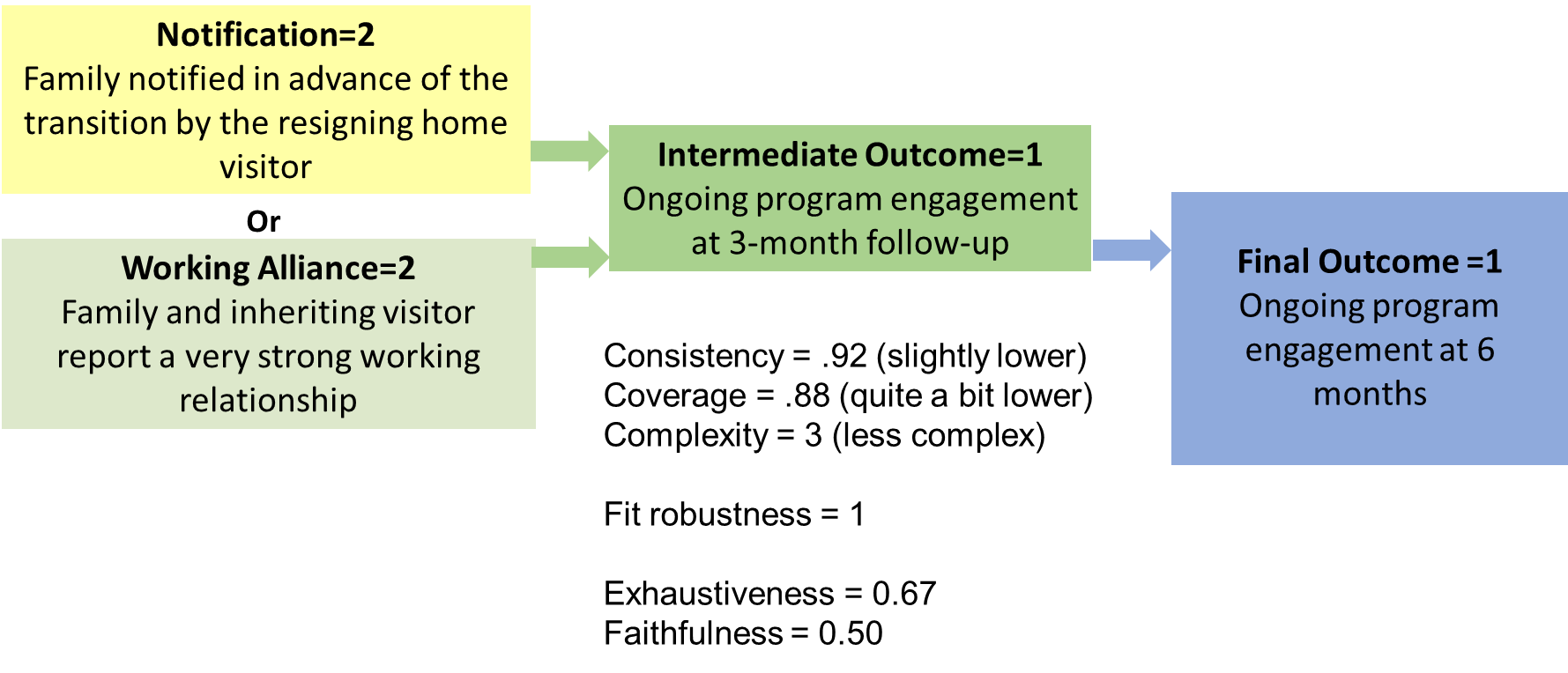


Because the most robust model is a submodel of the “stand out” model (which happened to be the second most robust model), this means the two models share many similarities and no contradictions. In the section below we explain the similarities and differences between these two models.

Both the initial “stand out” model and the most robust model include a ‘causal chain’ whereby the presence of certain conditions almost always lead to ongoing family engagement in the program 3 months following home visitor resignation; and ongoing engagement at 3 months almost always results in ongoing engagement at 6 months (with all but two families engaged at 3 months remaining engaged at 6-months). In the first part of this ‘causal chain’, the two conditions that independently make a difference for ongoing family engagement at 3 months were: 1) having received advance notification of the transition by the resigning home visitor or 2) having a strong positive working alliance with the inheriting home visitor at 3 months. A total of 26 families had one or both of these conditions and 25 of them remained engaged at the 3-month follow-up. This first causal chain in the complex solution was therefore highly consistent as evidenced by only 1 family who was engaged at 3-months despite the absence of both advance notification and strong positive working alliance with the inheriting home visitor.

In the second most robust model (which was the original, “stand-out” model), an additional path was identified to explain why three families, that were not covered by the most robust model, re-engaged at 6-months even though they were not engaged at 3-months (Figure 3). This second path to engagement at 6-months was the occurrence of another change in home visitor between the 3- and 6-month follow-up. This second path was consistent because all eight families who experienced another home visitor change between 3 and 6 months were engaged at 6-months (including the 3 who re-engaged). Despite the initial appeal of this model, the more robust and simpler model (shown in Figure 4) was favored for two main reasons: 1) We were concerned that the second most robust model (shown in Figure 3) may be overfit and 2) we hesitated to conclude that a change in home visitor contributes to ongoing engagement in the program (given that prior data suggested a home visitor change could contribute to families dropping out of the program).

There were an additional four models that seemed plausible and are mentioned as part of the limitations section in the main manuscript. One key difference with a few of these models is that instead of advance notification by resigning home visitor (N) being a path to engagement at 3 months, debrief (D) replaced this as a path within the first part of the causal chain.

Figure 5 illustrates the Boolean notation for the six most robust/feasible models. Other models are not shown because they either had much lower fit robustness scores and/or they were clearly overfit. Although models 7 and 8 are not shown below, their fit statistics were included to demonstrate how overfitted they are (as indicated by how much higher their complexity scores are compared to other “good fitting” models that explain the same outcome values).

**Figure 5.** Boolean solutions for the six most robust models with comments (in blue) and fit measures for the top eight models.


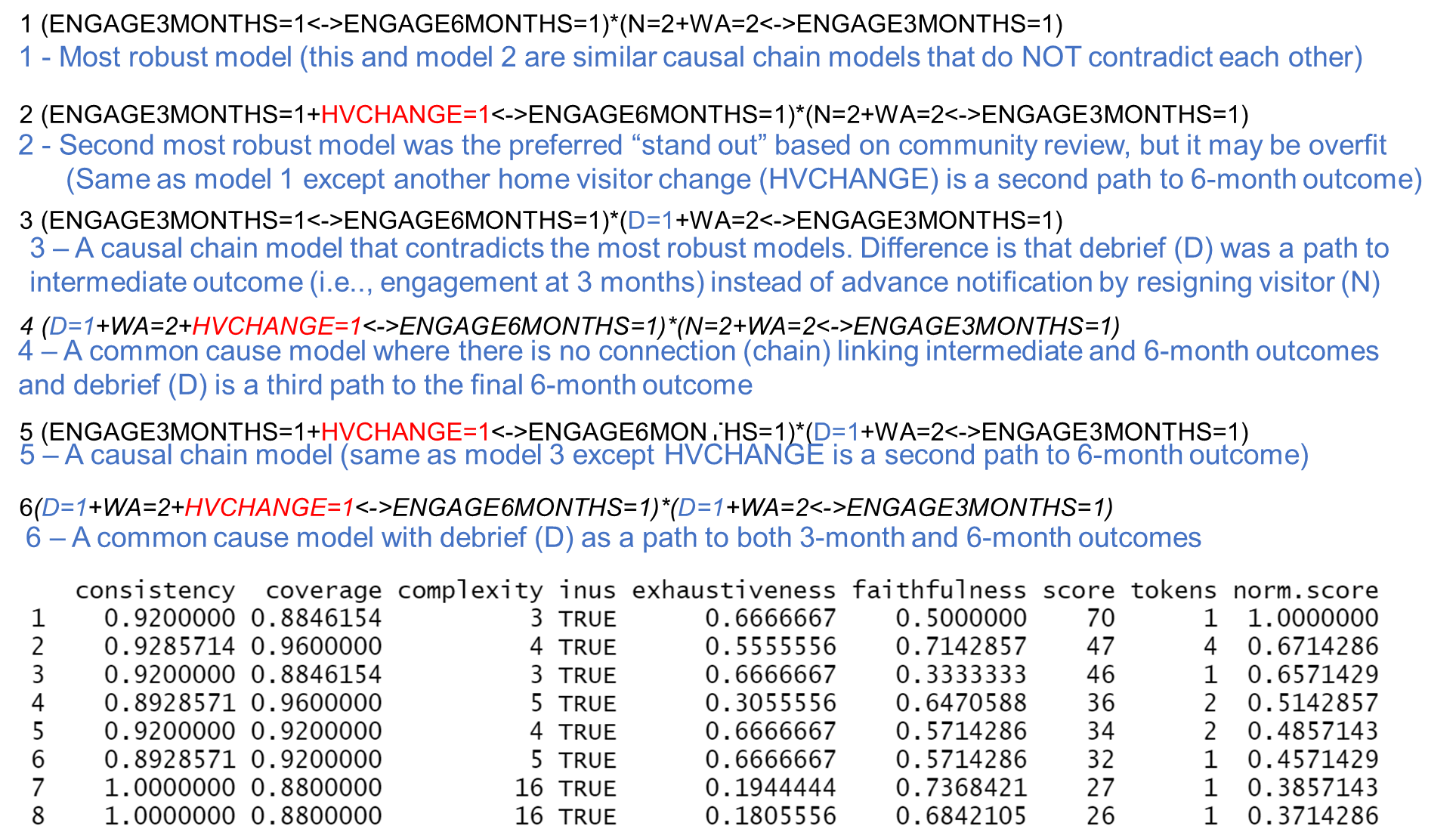


***Considerations Related to CNA***

There are certain considerations worth noting when using CNA. First, when model ambiguity is found we can never be certain which model is actually “correct”. However, as shown above, the top most robust models share multiple similarities. For instance, all the top models show that families with a very strong working alliance with the new home visitor almost always remain engaged at 3 months. The top two models both suggest that advance notification by the resigning home visitor is another independent protective condition that helps ensure ongoing engagement among families. However, models 3, 5 and 6 suggest that the resigning and inheriting home visitor debrief is the second protective condition leading to ongoing engagement (rather than advance notification).

Although the longitudinal nature of the study and use of CNA to identify redundancy free solutions are strengths, caution should be taken when making causal assertions in the absence of a randomized controlled trial. Finally, data fragmentation (which occurred because several possible configurations are not represented in the data set) can result in incomplete models (Baumgartner & Ambühl, 2018). Thus, the possibility remains that additional factor values (not identifiable from the current data set) may also be contributing to ongoing engagement.
